# An On-Line Low-Cost Irradiance Monitoring Network with Sub-Second Sampling Adapted to Small-Scale PV Systems

**DOI:** 10.3390/s18103405

**Published:** 2018-10-11

**Authors:** Manuel Jesús Espinosa-Gavira, Agustín Agüera-Pérez, Juan José González de la Rosa, José Carlos Palomares-Salas, José María Sierra-Fernández

**Affiliations:** Research Group PAIDI-TIC-168: Computational Instrumentation and Industrial Electronics (ICEI), University of Cádiz, EPSA, Av. Ramón Puyol S/N, Algeciras, 11202 Cádiz, Spain; manuel.espinosa@uca.es (M.J.E.-G.); juanjose.delarosa@uca.es (J.J.G.d.l.R.); josecarlos.palomares@uca.es (J.C.P.-S.); josemaria.sierra@uca.es (J.M.S.-F.)

**Keywords:** irradiance monitoring network, wireless sensor network, very short-term solar forecasting, microgrids, pyranometer, lux-meter, cloud enhancement

## Abstract

Very short-term solar forecasts are gaining interest for their application on real-time control of photovoltaic systems. These forecasts are intimately related to the cloud motion that produce variations of the irradiance field on scales of seconds and meters, thus particularly impacting in small photovoltaic systems. Very short-term forecast models must be supported by updated information of the local irradiance field, and solar sensor networks are positioning as the more direct way to obtain these data. The development of solar sensor networks adapted to small-scale systems as microgrids is subject to specific requirements: high updating frequency, high density of measurement points and low investment. This paper proposes a wireless sensor network able to provide snapshots of the irradiance field with an updating frequency of 2 Hz. The network comprised 16 motes regularly distributed over an area of 15 m × 15 m (4 motes × 4 motes, minimum intersensor distance of 5 m). The irradiance values were estimated from illuminance measurements acquired by lux-meters in the network motes. The estimated irradiances were validated with measurements of a secondary standard pyranometer obtaining a mean absolute error of 24.4 W/m${}^{2}$ and a standard deviation of 36.1 W/m${}^{2}$. The network was able to capture the cloud motion and the main features of the irradiance field even with the reduced dimensions of the monitoring area. These results and the low-cost of the measurement devices indicate that this concept of solar sensor networks would be appropriate not only for photovoltaic plants in the range of MW, but also for smaller systems such as the ones installed in microgrids.

## 1. Introduction

Sensor networks are consolidating as promising data sources for future solar energy forecasting applications, particularly for local and very-short term applications. At these scales, solar instrumentation is focused on capturing the cloud motion, which is the main cause of irradiance variations. All-sky cameras and satellite imagery have been used for this purpose, but, due to the complexity and inaccuracies associated with the image-to-irradiance conversion process, solar sensor networks are gaining attention as a direct solution to capture the local irradiance field and its evolution [1]. This trend has been reinforced by the recent proliferation of meteorological and energy sensors and the developments in big data applications for smart energy management based on information sharing among multiple systems. In this sense, Zhou et al. pointed to microgrids (MGs) as important emerging sources of solar measurements and main beneficiaries of solar forecasts derived from big data techniques [2]. Furthermore, MGs are considered to be key elements in the large-scale grid integration of solar energy since they are efficient in mitigating the negative effects associated with the intermittent PV generation. Forecasting tools specifically designed for MG energy management systems would be valuable for this purpose. However, Mazzola et al. indicated that, in small systems such as MGs, “the cost of the forecast service plus other automation infrastructure may not be economically justified” [3].

Solar sensor networks have been used in different studies, generally involving scales larger than the ones associated with MGs. Data are acquired by a set of sensors randomly distributed over the concerned area, and later processed to estimate the solar variability of the area or forecast the irradiance at a certain point. For instance, Gutierrez-Corea et al. used artificial neural networks to forecast the irradiance in a target station analyzing data from 30 stations within a radius of 100 km and a temporal resolution of 15 min [4]. Supported by 13 solar sensors distributed over an area of 20 km × 40 km, Lorenzo et al. used different persistence methods to forecast irradiance. The sensors included pyranometers and photodiodes which provided 1-s resolution data with a latency of 1 min [5]. The solar sensor network described in [6] covered a similar area, although, in this case, it comprised 24 stations more homogeneously distributed which provided data with a resolution of 1 min. The study has added value, as it is focused on the optimal design of a solar sensor network, which is a topic rarely addressed in solar research literature.

The local scale of MGs demands solar sensor networks with characteristics different to the ones described above. The main challenge is associated with the drastic variations of the PV generation produced by the clouds, which have a lower impact in larger power systems as a result of the well-known spatial smoothing effect [7]. In [8], it is concluded that reliable descriptions of the clouds required 625 measurement points over an area of 2.5 km × 2.5 km and temporal resolution of 10 s. The NREL radiometer grid of Hawaii, USA represents a good example of the applications of solar information at these spatial and temporal scales. The NREL Radiometer grid was composed of 17 LICOR LI-200 pyranometers distributed over an area of 1 km × 1 km. (The minimum, median and maximum intensesnsor distances were 86, 381 and 1130 m, respectively [9]). Solar radiation was acquired with a resolution of 1 s and syncronized by GPS receivers. The versatility and usefulness of these data are corroborated by their utilization in forecasting applications [9], variability characterizations [10] and instantaneous irradiance estimations [11]. In [12], the solar sensor network of Melpitz, Germany was composed of 50 silicon photodiode pyranometers distributed over an area on 2 km × 2 km, achieving a maximum density in a central area of 0.5 km × 0.5 km where 25 pyranometers were installed (The shortest intersensor distances ranged from 28 m to 51 m [12]). Data were acquired at 10 Hz, stored as 1-s averaged values and subsequently analyzed to determine the irradiance variability and the spatial smoothing effects for PV-power integration. The NextEra Project deployed a grid of 48 pyranometers, each of them associated to a data-logger recording 1-s irradiance data, distributed over one square-mile in Flagstaff, Arizona. It is concluded that four stations would be enough to reasonably estimate the solar resource variability at a given site. The irradiance measurement network at the Plataforma Solar de Almería, Spain comprised 20 pyranometers distributed over 350 m × 280 m measuring at 1 Hz. The network design and its utilization for detecting cloud motion are described in [13].

Even if these studies deal with the local irradiance field, the concerned monitoring areas are significantly larger than the ones available for PV systems in MGs or nanogrids, commonly installed on building rooftops. The necessity and characteristics of solar data specifically focused on these systems are outlined in some studies. Lohmann et al. explained how smaller PV systems require more spatiotemporal resolution [12] and Scolari et al. pointed that MG energy management systems would be benefited by solar information in the sub-second range [14], but Torregrossa et al. stressed that little research has been done in this aspect [15]. In [16], the acquisition of this information is considered a main challenge to reinforce the solar forecasts used by MG energy management systems. To the authors’ knowledge, in the scientific literature, there are no examples of irradiance monitoring networks with dimensions of tens of meters and sub-second sampling.

For a proper design of irradiance monitoring networks with sub-second sampling, the sensors’ time response must be carefully considered. Typically, the time response of the pyranometers ranges between 5 and 20 s, thereby acting as a low-pass filter for sub-second irradiance variations that affects the PV generation. In [17], Yordanov et al. compared the response of a pyranometer and a PV cell, showing how the pyranometer was not able to capture the peaks and fast variations of irradiance observed in the PV cell. These peaks and variations are mostly related to over irradiance events produced by the clouds’ edges that can last from seconds to minutes, reaching peaks up to 2000 W/m${}^{2}$ and requiring sampling frequencies of 1 Hz or higher to be accurately captured. Tapakis and Charalambides also studied these events, including a discussion of their effects in PV-inverters [18]. In this sense, it is asserted that the over irradiance events can produce irreparable damages to inverters, and indicated that they could be prevented by supplying high frequency measurements to the MPPT devices. Thus, both studies [17,18] emphasized the necessity of sensors able to work in these irradiance and temporal ranges to improve the PV-inverters operation. Lux-meters can be an interesting solution regarding time response and investment in these cases. Schenk et al. considered lux-meters in preliminary design stages of the irradiance network at the Plataforma Solar de Almería, Spain, reporting promising results in some cases and anomalous behaviors in others [13]. The results of these studies are further discussed below.

This paper deals with the design and test of a solar sensor network adapted to the necessities of small-scale PV systems. According to the above considerations, the network should provide on-line, sub-second measurements and an appropriate spatial resolution to support very short-term forecasting applications. The network should also be able to capture the over irradiance events produced by the clouds’ edges to prevent damages in PV-inverters. Additionally, it should demand an easy-to-implement infrastructure and a low investment appropriated to the scale of the concerned systems. In this sense, wireless communications are chosen to minimize installation problems and costs. The paper is structured as follows. Section 2 describes the motes and the different elements involved in their design and construction. Section 3 explains the calibration process to adjust the illuminance-to-irradiance conversion, and providing a characterization of the accuracy of the measurements. Section 4 focuses on the configuration of the network, and Section 5 and Section 6 contain the description of the results and conclusions, respectively.

## 2. Motes description

**Figure 1:**
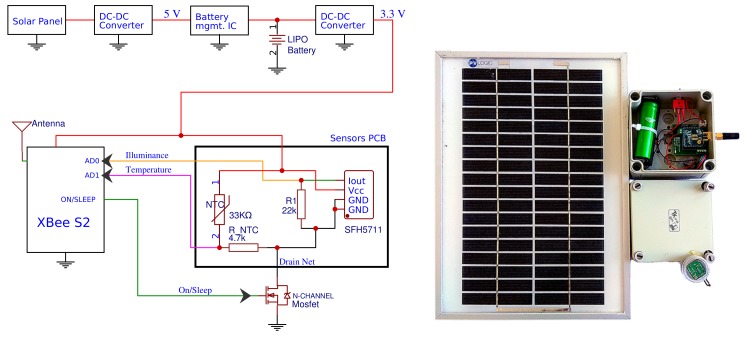
Mote schematics (**left**); and hardware prototype (**right**).

The WSN is composed of motes that basically comprise a solar sensor and a communication module powered by a battery and a PV panel to assure autonomous operation (Figure 1). As stated in [19], the two main components that affect the power consumption in WSN motes are the microcontroller and the radio module. To minimize the cost and power consumption, the design effort has been focused on developing motes without external microcontroller. As explained in [20], this concept also increases reliability (lower number of elements and interactions), flexibility (reconfigurations are easier and faster) and data rates (bottlenecks due to the external microcontroller are avoided) in the deployed networks. Note that these properties are critical in the present case, since simplicity and high data rates must be primary characteristics of solar sensor networks at local scale.

Regarding the environmental variables, irradiance is inferred from illuminance measurements provided by SFH 5711 sensors. This sensor is analogous to SFH 5712 which has been previously used in solar energy applications [13,21]. SFH 5711 differs from SFH 5712 in the output mode. The first has a logarithmic current output while the second has an I2C interface to read the measurement. Both are widely used in commercial devices as a sunlight sensor and precise lux-meter. Finally, a NTC termistor, ND03N00103K from AVX, is used for temperature measurements.

The communication is based on ZigBee/IEEE 802.15.4 protocol. Concretely, the XB24CZ7SIT-004 XBee module (included in XBee S2 Series RF modules) has been used, which a priori fulfills the network requirements of intersensor distances, data rates and investment. The selected XBee module support two working modes: AT and API. AT mode (or transparent mode) works like a wireless UART communication. On the other hand, API (Application Programming Interface) mode requires a specific UART data structure. API mode allows accessing the I/O interface and the subsequent parameter modification without entering the command mode and, thereby offering advantages over the AT mode (The API data structure can be consulted in [22]).

The main PCB holds the circuitry for interconnection among elements, including a battery management integrated circuit for safe charging of the Li-Po battery (2300 mAh, 3.7 V), a switching voltage regulator to power up the radio module and the signal conditioning circuitry and a N-channel MOSFET used as switch to power up the sensors PCB only when is needed, avoiding unnecessary battery drain. The radio module is also assembled to the main PCB, and both allocated inside an IP66 box together with the Li-Po battery. A power switch is added to the box for manual activation. The sensor PCB is placed inside a transparent case glued and sealed with silicone to the upper side of the IP66 box to allow direct light measurements. Finally, the PV solar panel (5 Wp) is also situated outside the box.

The total cost of each mote is below 5% of the cost of a secondary standard pyranometer.

## 3. Calibration

An inference process is necessary to efficiently estimate irradiance values from the illuminance measurements provided by the lux-meter. The accuracy in these estimations determine the validity of the network for solar irradiance monitoring. For this purpose, a secondary standard pyranometer (CMP11 Kipp & Zonen) has been used as reference. Table 1 summarizes the main characteristics of the pyranometer and lux- meters (More information about the pyranometer and lux-meter can be found in [23,24]).

**Figure 2:**
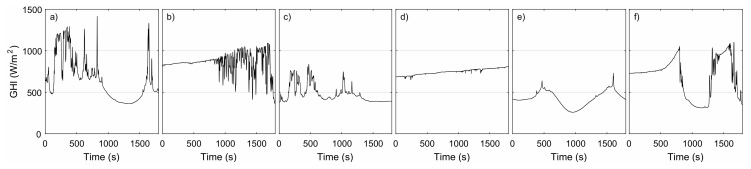
Six examples of real irradiance profiles used for calibration. The starting dates and times expressed in UTC+2 in each case: (**a**) 7 June 2018 13:20:42; (**b**) 8 June 2018 11:21:49; (**c**) 9 June 2018 09:48:22; (**d**) 9 June 2018 10:44:52; (**e**) 10 June 2018 12:35:15; and (**f**) 11 June 2018 11:00:46.

The pyranometer and 16 motes were closely installed in the rooftop of the Polytechnic School of Engineering in Algeciras, Spain (Latitude: 36.12${}^{\circ }$ N; Longitude: 5.45${}^{\circ }$ W), conforming a network similar to the one described in Section 4. Thus, the calibration conditions were similar to those of the real operation of the monitoring system. Data from a total of 12 h were used for calibration. These data were acquired at 2 Hz in periods of 30 min within a week (5–11 June 2018) trying to capture different meteorological situations associated with different degrees of cloud cover. Figure 2 shows pyranometer data of six 30-min periods as representative examples of the calibration conditions. Note that in some cases the irradiance values are clearly above the expected maximum values under clear sky conditions, which is approximately 1000 W/m${}^{2}$. These values can be explained as over irradiance events produced by the clouds’ edges.

**Figure 3:**
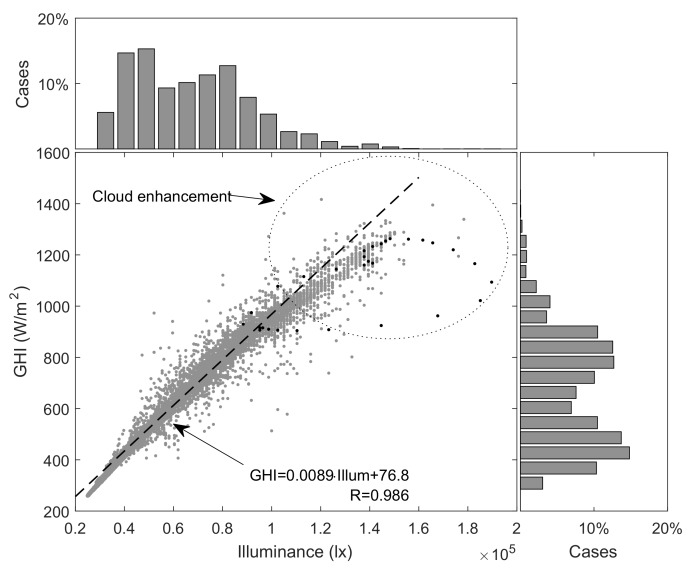
Scatter plot and histograms associated with the pyranometer and lux-meter #6 associated with the whole period used for calibration.

**Figure 4:**
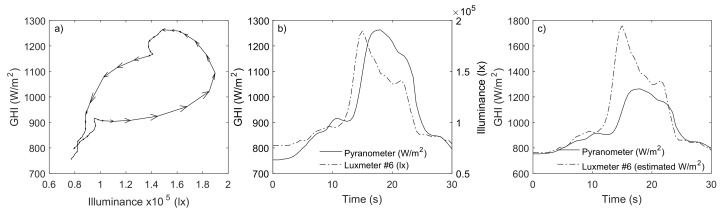
Analysis of the pyranometer and lux-meter #6 performances during a cloud enhancement event: trajectory in the scatter plot (**a**); comparison of measured illuminance and irradiance in the time domain (**b**); and comparison of estimated and measured irradiance in the time domain (**c**).

Once the data from the pyranometer and the motes were acquired, an individual calibration of each mote was performed. Since data showed excellent correlation in all cases—correlations coefficients (R) ranged from 0.975 to 0.991—the illuminance-to-irradiance conversion was adjusted by linear regression. Thus, a linear model was associated with each node to perform individualized irradiance estimations. Figure 3 shows one representative case involving a correlation coefficient R = 0.986. The figure includes the corresponding regression line and its equation. Data are well-fitted by regression line for most of the measurement range, although certain deviation can be observed below 400 W/m and above 1200 W/m. Enclosed in a circle, there are points that can be associated with the already commented over irradiance events. These points represented less than 2% of the registers. Histograms at the top and right side of the scatter plot illustrate about the measurements distribution. The black dots are used to remark the detail represented in Figure 4, which is discussed below. Overall, the scatter plots of the resting 15 motes present similar features. Table 2 details the results for each mote.

The scatter plots used in the calibration process present peculiar curves that, to a greater or lesser extent, are responsible for a relevant part of the dispersion observed between the pyranometer and mote registers. These curves were also reported by Schenk et al. and interpreted as anomalous behavior of some lux-meters. The result was the exclusion of these sensors from their solar sensor network despite the promising results in some cases and the low investment costs [13]. These anomalous curves can be produced by the different time response between the pyranometers and lux-meters: the former in the range of seconds and the latter in the range of milliseconds. To illustrate, Figure 4a shows one of these curves extracted from the scatter plot of Figure 3. The arrows indicate the sequence of the 61 selected points, corresponding to a period of 30 s (sampling frequency of 2 Hz). Figure 4b reproduces the concerned GHI and illuminance measurements in the time domain. The selected interval covers a moment of clear sky between clouds. Figure 4b shows how the lux-meter captured the drastic changes approximately two seconds before the pyranometer reaction. Thus, in a first step, there is an increase of luxes without increase of irradiance, which produces a horizontal trajectory in the scatter plot (Figure 4a). As pyranometer reacts, the trajectory becomes more vertical. Following the sequence of measurements, the entire curve can be reproduced. Finally, as the values remain stable due to the presence of the new cloud, the points converged to the initial ones. Besides the commented “delay”, Figure 4b also shows how the lux-meter measurements present more details, while the pyranometer measurements are smoother. The illuminance-to-irradiance conversion described above allows to compare the performance of both sensors (lux-meter #6 and pyranometer) in terms of irradiance, as represented in Figure 4c. The longer time response of the pyranometer acts as a low-pass filter that smooths fast irradiance variations, thereby omitting irradiance events that occurs in shorter time scales. The lux-meter is able to capture these rapid changes, registering remarkable features as irradiance peaks 50% higher than the ones measured by the pyranometer. Under stable conditions or slow variations of irradiance, both sensors provide similar measurements. These results indicate that lux-meters can provide measurements more representative of the PV-cell behavior in the sub-second range, thereby being even more suitable than pyranometers for monitoring systems at these scales.

**Figure 5:**
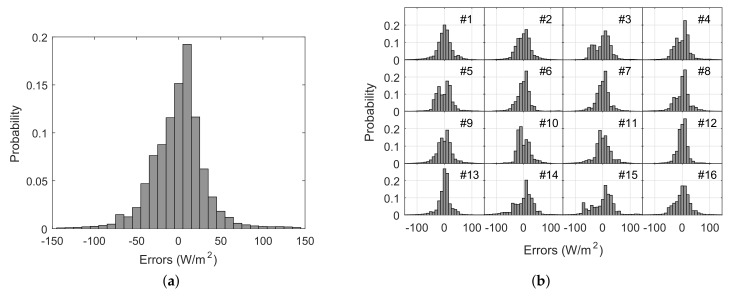
Aggregated error distribution including the 16 motes (**a**); and error distributions for each mote according to the network layout (**b**).

Figure 5a shows the error distribution resulting from the aggregation errors from the 16 motes, which provides a general characterization of the expected accuracy in these sensors. The error distribution presents a standard deviation of 36.1 W/m${}^{2}$, being the 95% of errors within a range of 68.1 W/m${}^{2}$. The mean absolute error is 24.4 W/m${}^{2}$. Figure 5b and Table 2 shows the individualized results for each mote. It is worth mentioning that these errors provide a conservative characterization of the mote’s performance, since they include the commented deviations derived from the time response that, in some sense, can be interpreted as pyranometer’s errors. Anyway, for characterization purposes, the pyranometer data have been considered as an exact reference. In this sense, the observed errors also compute the effects of all the uncertainty sources that affect the measurement chain: data acquisition strategy, transmission problems or different performances under different atmospheric conditions. The obtained uncertainty is thereby a characterization of the performance of the motes and the monitoring system as a whole. A deeper analysis on the effects of the different uncertainty sources would be matter of future work.

## 4. Network Configuration

XBee modules are thought to build mesh networks. In a first stage, the mesh architecture was considered and the motes were configured to work as measurement devices and routers simultaneously. In these conditions, we found problems to increase the sampling frequency above 1 Hz. Furthermore, the network performance was highly unstable and similar experiments provided very different results. The reception of packets was irregular among the 16 motes and variable in time, making impossible the implementation of an irradiance monitoring network for the proposed objectives. Thus, the router role was disabled and the motes were set as measurement end devices only, showing an important improvement in their data acquisition properties. However, this configuration also required the inclusion of additional devices acting as routers to link the motes and the coordinator, which in turns transmitted the information to the PC through a serial port. The routers were identical to the motes but removing the sensors and the associated circuitry. With this configuration, the packets followed a mote–router–coordinator path, giving uniformity to the information flow from each mote.

**Figure 6:**
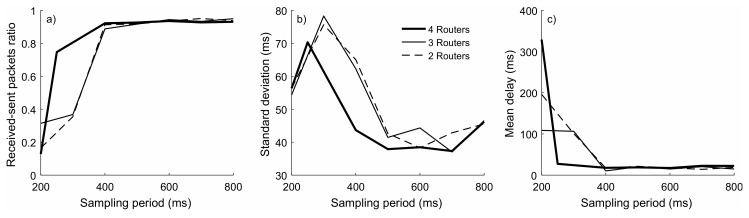
Network performance under different configurations: (**a**) Received-sent packets ratio, (**b**) Standard deviation and (**c**) Mean delay in the reception of packets.

To determine the network optimal operation conditions, a series of tests were performed involving different sub-second sampling periods and different numbers of routers. Figure 6 summarizes the results of these tests. The characterization of the network operation was based on three factors:

- The ratio of successfully received packets, as the relation between the received packets and the packets that have been sent according to the established sampling period (see Figure 6a): A ratio of 1 involves that all sent packets were received.
- The temporal dispersion of the received packets, quantified by the standard deviation in the time between packets (see Figure 6b): A low standard deviation is associated with a high periodicity, indicating a stable flow of information from the motes to the coordinator.
- The mean delay in the reception of packets, as the difference between the motes sampling period and the actual mean period between received packets (see Figure 6c): The mean delay is associated with the computation time and possible transmission delays, which are added to the sampling period of each mote.

The ideal situation would be associated with a ratio of successfully received packets of 1 and a standard deviation and mean delay of 0 ms in the time reception of packets. This would imply that all sent packets are received with a regular periodicity equal to the selected sampling period. The results in Figure 6 show that sampling periods shorter than 400 ms entail an important percentage of lost packets that obviously introduces dispersion and delay in the reception of packets. With longer sampling periods, the network became stable and the performances including two, three or four routers were similar, with the received-sent packet ratios close to the unity and mean delays around 20 ms in all cases. The presence of four routers only seems to slightly benefit the regular reception of packets.

Attending to these results, the final network configuration had the following characteristics:

- Sixteen motes act as end devices distributed on a regular grid of 15 m × 15 m (minimum intersensor distance of 5 m).
- Four routers channel information to the coordinator. The network was initialized by linking four motes to each router, but momentarily variations of this configuration were observed. Despite the minimum improvement associated with four routers, the redundancy of nodes ensured a more relaxed network operation and robustness against possible disconnections without a significant increase of costs.
- A sampling period of 480 ms was established. Thus, the addition of 20 ms related to the observed delays results in an actual sampling period of 500 ms (2 Hz).

## 5. Results

The estimation and final representation of the irradiance field are performed on a PC that receives the information packets from the motes through a serial port via the coordinator. The buffer is read every 500 ms and the received packets are individually processed. The illuminance measurements within these packets are converted to irradiance values by applying the specific linear model associated with each mote that sent each packet (see Section 3). Then, the irradiance values are represented in a 4 × 4 matrix according to the spatial position of each mote. Thus, the representation of the irradiance field is updated every 500 ms (Even though measurements from all motes are refreshed every 500 ms, the acquisition of data is not synchronized. The uncertainty derived from the de-synchronization is included in the errors evaluation in Section 3, as the calibration process was performed under measurement and communication conditions similar to those of the real operation of the system). Due to the temporal dispersion of packets—around 40 ms (see Section 4)—in some cases, two packets from the same mote can be received in a 500-ms interval. In these cases, the last received packet is selected to represent the most updated information. Similarly, there are 500-ms intervals in which no packet has been received from a certain mote. Then, the value of the concerned mote in the previous interval is maintained.

Once the network was installed and configured, several tests were carried out to verify the system’s ability in capturing the cloud motion. As preliminary results, the tests confirmed the stability of the network and the normal operation according to the design specifications. It was also corroborated that the motes’ energy demand was satisfied by the PV panel (5 Wp), thus assuring autonomy (The state of charge of the batteries never was below 60%).

**Figure 7:**
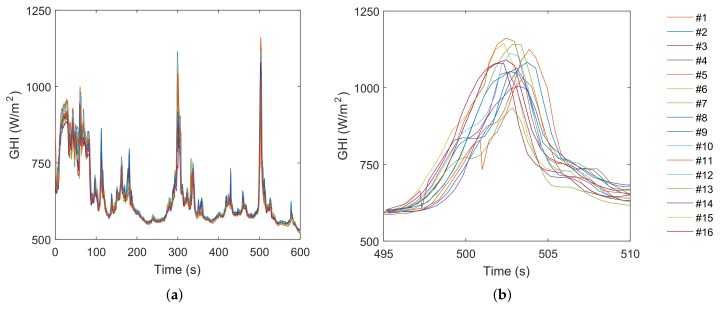
Estimated irradiance from the 16 motes: 10-min period (**a**); and 15-s detail (**b**).

**Figure 8:**
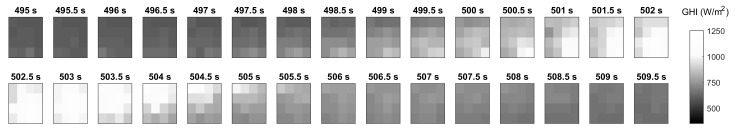
Irradiance field sequence of 15 s.

Figure 7a shows the estimated irradiances associated with the 16 motes in a period of 10 min (600 s). The measurements show consistency and illustrate about the general performance of the network under variable irradiance conditions. However, this information must be analyzed in the sub-second range to certify its utility in very short-term forecasts for small scale PV systems. In this sense, the right figure details the most significant irradiance variation in this period: a cloud enhancement event occurred between the Seconds 495 and 510. It can be observed how the event is clearly registered by all motes but with different intensity and slight temporal shift. For a better interpretation of these data, Figure 8 represents the sequence of the event according to the spatial position of each mote in the grid. This visualization clearly reveals the advance of the clouds in NW direction, which explains the temporal shift observed in the motes measurements. The sequence confirms that the sampling frequency and the density of measurement points were able to capture the cloud motion at these temporal and spatial scales.

**Figure 9:**
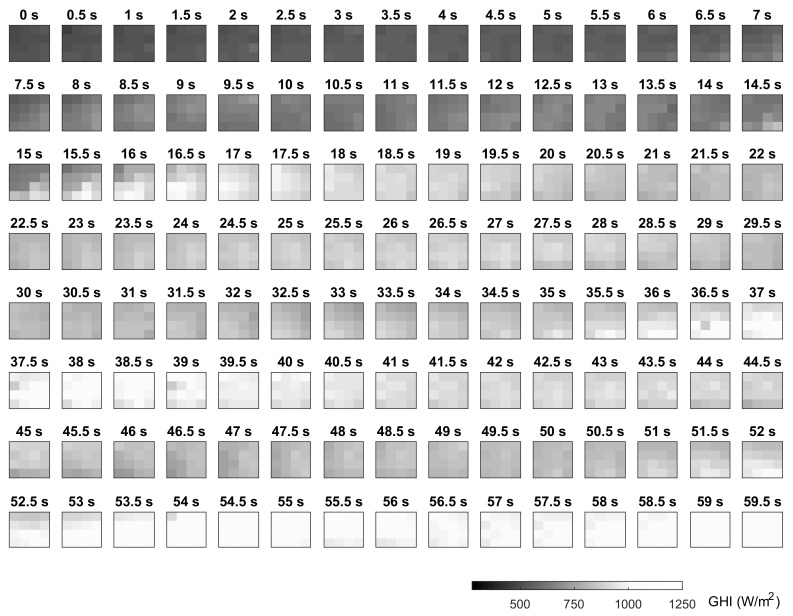
Irradiance field sequence of 60 s.

Figure 9 shows a 60-s sequence to give an idea of the continuous operation of the system. The sequence includes different situations from cloudy to clear conditions. Again, the cloud movement towards the NW can be observed. Some spatial features of the irradiance field can also be observed. For instance, during Seconds 15–18, a high irradiance zone passed over the monitoring area but mainly affecting the motes placed in the SW. The information provided by the sensor network can be a valuable input for very short-term forecasting applications. For instance, machine-learning techniques can be easily implemented to determine the current cloud velocities, and on this basis generate predictions for a central point (the PV panels) according to the irradiance variations observed at the outer motes.

Finally, it is worth mentioning that the motes were able to work autonomously entering in sleep mode during the nights only, indicating that the energy supply provided by a 5 Wp panel was enough for the motes and routers operation.

## 6. Conclusions

The present study described and tested an irradiance monitoring system adapted to the restrictive necessities of MGs or small-scale PV systems. These necessities can be summarized as sub-second sampling frequency, high density of measurement points and a low investment. The monitoring system was designed as a wireless sensor network based on the ZigBee protocol comprising 16 motes regularly distributed over an area of 15 m × 15 m. The irradiance values were inferred from illuminance measurements acquired by lux-meters installed in each mote. After the calibration process, the inferred irradiance values showed a mean absolute error of 24.4 W/m${}^{2}$ and a standard deviation of 36.1 W/m${}^{2}$ with respect to the measurements of a secondary standard pyranometer used as reference. The sampling frequency was set at 2 Hz since higher frequencies produced instability in the network operation worsening the information flow. In these conditions, the monitoring system was able to capture the irradiance variations associated with the cloud motion with enough temporal and spatial resolution to outline the main features of the irradiance field and its evolution, including over irradiance events produced by the clouds’ edges. The results indicate that monitoring systems with these characteristics could provide excellent support for very short-term forecasting applications that benefit the operation of small PV systems with a low investment, and consequently also applicable to larger systems.

Collaterally, the effect of the pyranometer’s time response in smoothing the rapid irradiance variations was also observed, indicating that lux-meters are probably a better option for detailed sub-second irradiance measurements. The difference between both sensors was clear during cloud enhancement events.

## Figures and Tables

**Table 1:** Specifications.

	CMP11	SFH 5711
Measurement Range	0 to 4000 W/m${}^{2}$	3 to 80 klx (*)
Spectral range	285 to 2800 nm	475 to 650 nm
Sensitivity	7.66 μV/W/m${}^{2}$ (**)	S = 10 μA/dec (***)
Response time	<5 s	<0.1 ms
Field of view	180${}^{\circ }$	180${}^{\circ }$

(*) Range in which the logarithmic relation is assured; (**) From the calibration document provided with the pyranometer; and (***) $I_{out}=S\cdot log\left(E_{v}/E_{0}\right)$ where $E_{v}$ is the input illuminance and $E_{0}$ = 1 lx.

**Table 2:** Motes.

	MAE (W/m${}^{2}$)	SD (W/m${}^{2}$)	R		MAE (W/m${}^{2}$)	SD (W/m${}^{2}$)	R
Mote #1	24.4	40.4	0.983	Mote #9	25.2	39.9	0.983
Mote #2	24.8	38.4	0.984	Mote #10	21.1	27.6	0.990
Mote #3	26.9	36.9	0.986	Mote #11	25.6	40.9	0.982
Mote #4	24.8	39.0	0.984	Mote #12	16.0	25.4	0.991
Mote #5	25.3	36.5	0.983	Mote #13	18.5	30.0	0.991
Mote #6	20.7	32.8	0.986	Mote #14	32.2	47.3	0.976
Mote #7	20.2	29.3	0.991	Mote #15	35.3	48.6	0.975
Mote #8	23.7	38.8	0.984	Mote #16	25.6	38.6	0.984

## Abbreviations

The following abbreviations are used in this manuscript:

GHI	Global horizontal irradiance
MAE	Mean absolute error
MG	Microgrid
MPPT	Maximum power point tracking
PCB	Printed circuit board
PV	Photovoltaic
PC	Personal computer
SD	Standard deviation
